# Immunonutrition in Radical Cystectomy: State of the Art and Perspectives

**DOI:** 10.3390/cancers15143747

**Published:** 2023-07-24

**Authors:** Amanda Casirati, Valentina Da Prat, Arianna Bettiga, Lucia Aretano, Francesco Trevisani, Emanuele Cereda, Alberto Briganti, Elisa Colombo, Giorgia Preziati, Francesca De Simeis, Andrea Salonia, Francesco Montorsi, Riccardo Caccialanza, Richard Naspro

**Affiliations:** 1 Clinical Nutrition and Dietetics Unit, Fondazione IRCCS Policlinico San Matteo, 27100 Pavia, Italy; a.casirati@smatteo.pv.it (A.C.); v.daprat@smatteo.pv.it (V.D.P.); e.cereda@smatteo.pv.it (E.C.); eli.colombo@smatteo.pv.it (E.C.); g.preziati@smatteo.pv.it (G.P.); f.desimeis@smatteo.pv.it (F.D.S.); 2 Department of Urology and Division of Experimental Oncology, IRCCS San Raffaele Scientific Institute, Vita-Salute San Raffaele University, 20132 Milan, Italy; bettiga.arianna@hsr.it (A.B.); trevisani.francesco@hsr.it (F.T.); briganti.alberto@hsr.it (A.B.); salonia.andrea@hsr.it (A.S.); montorsi.francesco@hsr.it (F.M.); 3 Urology Unit, Fondazione IRCCS Policlinico San Matteo, 27100 Pavia, Italy; l.aretano@smatteo.pv.it (L.A.); r.naspro@smatteo.pv.it (R.N.)

**Keywords:** immunonutrition, bladder cancer, cystectomy, nutritional status

## Abstract

**Simple Summary:**

Preoperative nutritional status is a pivotal aspect to consider in cancer patients undergoing radical cystectomy, as malnourished individuals are more prone to post-surgical complications. The loss of muscle mass is a significant consequence of cancer-related malnutrition and is associated with increased risks of readmission, longer hospital stays, and higher mortality rates. This narrative review explores the concept of “immunonutrition”, which consists of the use of specific nutrients to boost the immune system and improve postoperative outcomes. By reviewing existing scientific literature, promising evidence was found that supports immunonutrition in reducing complications, including infections, after bladder surgery. These findings highlight the need for further research to determine the optimal approach, regardless of nutritional status, for improving patient outcomes after bladder surgery. The development of uniformly designed randomized controlled trials is necessary to establish the most effective dosage, timing, and duration of perioperative immunonutrition and to confirm the available preliminary evidence.

**Abstract:**

Preoperative nutritional status is a pivotal aspect to consider in patients with cancer undergoing radical cystectomy (RC), as those at risk of malnutrition or already malnourished are more prone to post-surgical complications. The loss of muscle mass is a major consequence of cancer-related malnutrition. It is associated with increased risk of hospital readmission, longer hospitalization, and higher mortality. Nowadays, the close relationship between nutritional and immunological aspects under stressful conditions, such as surgery, represents an emerging scientific and clinical issue. Indeed, the synergistic action of reduced food intake and systemic inflammation generates metabolic derangements with tissue catabolism, including skeletal muscle breakdown, which is, in turn, associated with immune system dysfunction. In order to offer an additional immune-nutritional boost to the post-surgical phase, particularly in malnourished patients, nutritional support may include oral nutritional supplements and/or enteral formulas enriched with specific nutrients such as omega-3 fatty acids, arginine, glutamine, and nucleotides, with acknowledged immune-modulating effects. In the present narrative review, we addressed the state of the art of the available scientific literature on the benefit of immunonutrition in patients undergoing RC for cancer and suggest possible future perspectives to be explored. Although the role of immunonutrition was found to be little explored in the context of urologic oncology, the preliminary available data on radical cystectomy, summarized in the present paper, are promising and suggest that it may improve postoperative outcomes through immunomodulation, regardless of nutritional status before surgery.

## 1. Introduction

Radical cystectomy (RC) with urinary diversion and lymph node dissection is the standard treatment for muscle-invasive bladder cancer (MIBC), which is predominantly diagnosed in elderly patients [1,2]. RC is an extensive surgical procedure associated with an overall complication rate of 26–78% and a mortality rate of 1–4% [3,4]. Gastrointestinal complications are the most common (29%) and, among them, postoperative ileus occurs in up to 26% of patients, although a high incidence of infectious complications (25%) and wound-related complications (15%) has also been reported [3,5].

The guidelines on the treatment of MIBC stated that “Cystectomy patients at high risk for malnutrition should undergo nutritional counseling in preparation for surgery with the goal of optimizing nutritional status prior to surgery” [6]. The estimated prevalence of malnutrition in RC population ranges from 21% to 55% using the Nutritional Risk Screening (NRS) tool [7]. Of note, it has been demonstrated that patients at risk of malnutrition are more prone to post-surgical complications [8]. Moreover, nutritional status before RC is a strong predictor of 90-day mortality and poor overall survival [9].

Poor preoperative nutritional status may derive from the combined action of cancer itself, age-related frailty, and neoadjuvant chemotherapy effects, which negatively affect oral feeding. The synergistic action of reduced food intake and systemic inflammation generates metabolic derangements with tissue catabolism, including skeletal muscle [10,11].

A close relationship has been detected between nutritional and immunological aspects after stressful conditions such as surgery. Indeed, the loss of muscle is a long-term clinical consequence of the metabolic response to stress [12]. Muscle tissue plays a key role in this metabolic interplay, with amino acid metabolism being deeply affected by stress-related responses. First, plasma arginine levels rapidly decrease due to augmented myeloid-derived suppressor cells and proinflammatory cytokines generated by T-helper (Th) 1 and Th2 imbalance. Arginine deficiency can reduce nitric oxide, collagen, T-cell function and protein translation, leading to thrombosis, increased susceptibility to infection after surgery, wound breakdown, and muscle wasting. As arginine is a conditionally essential amino acid, improving its status restores the T-lymphocyte count [13]. Second, a rapid decrease in glutamine levels has been observed. Glutamine is a non-essential amino acid that represents the main source of energy for leukocytes and enterocytes and is involved in decreasing inflammation by regulating reactive oxygen species [13]. Skeletal muscle is closely related to the immune system mainly through glutamine production [14].

Loss of muscle mass is one of the main features of sarcopenia and often derives from cancer-related malnutrition. It is associated with an increased risk of hospital readmission, longer hospitalization, and higher mortality [15]. Chemotherapy contributes to skeletal muscle depletion in cancer patients. The adverse effects of antineoplastic agents, such as reduced appetite and early satiety, impact food intake and body weight. Studies have shown that patients with muscle mass loss experience higher toxicities from drugs like 5-fluorouracil, capecitabine, and sorafenib [16,17]. Patients with reduced muscle mass are more likely to require dose reductions, treatment delays and early termination, and exclusion from clinical trials, all of which negatively impact survival [16]. In murine models, the administration of FOLFIRI (5-FU, leucovorin, irinotecan) and FOLFOX (5-FU, leucovorin, oxaliplatin) leads to weight loss, adipose tissue loss, skeletal muscle wasting, weakness, and involves mechanisms such as the hyperactivation of catabolic signaling pathways, sarcomere structural changes, and the depletion of muscle mitochondria [17,18]. Although one study demonstrated that skeletal muscle change during neoadjuvant chemotherapy prior to RC is an independent predictor of ileus, infection, and other complications [19], conclusive data on the impact of sarcopenia in MIBC patients are not available due to limited evidence [20]. Conversely, the loss of skeletal muscle in metastatic urothelial bladder carcinoma has been demonstrated to be a significant predictor of 90-day mortality and postoperative complications [21].

With this background, early referral and proactive nutritional care should be considered on a routine basis. Nutritional support should be ideally initiated when patients are not yet malnourished; thus, the early evaluation of nutritional risk is mandatory. To prevent nutritional deficiencies, it is crucial to ensure that meals have adequate food composition. Nutrients obtained from the diet play essential roles in cellular function, energy supply, and immune defense. Proteins, in particular, are vital macronutrients for the immune system. Amino acids, the building blocks of proteins, have various functions within the immune system. They regulate the activation of adaptive and innate immune cells such as B cells, T cells, NK cells, and macrophages. Amino acids also contribute to lymphocyte proliferation and the production of antibodies, cytokines, and cytotoxic factors [22]. During the perioperative period, nutritional intake is essential to support increased nutritional needs resulting from the hypermetabolic and inflammatory state. When protein intake is insufficient, skeletal muscle becomes the primary source of essential amino acids necessary for maintaining overall protein synthesis in the body. Consequently, there is an elevated demand for dietary protein after surgery to reduce the risk of muscle catabolism [23]. The optimal supply for cancer patients has not been determined and the recommendations of the European Society for Clinical Nutrition (ESPEN) range between a minimum protein supply of 1 g/kg/day and a target supply of 1.2–2 g/kg/day, especially if inactivity and systemic inflammation are present [24].

The first type of nutritional support should be dietary counseling (DC) and oral nutritional supplement (ONS) administration when needed, aiming to fully satisfy nutritional requirements [25]. To counteract protein catabolism in the first phases after cystectomy, total parenteral nutrition (TPN) is widely used in the postoperative routine care regardless of nutritional status, gastrointestinal function, TPN-related increased economic costs, and infection complications risk [26,27]. However, early enteral nutrition (EEN) by oral intake or enteral feeding with a nasojejunal tube has been shown to result in reduced infectious complications compared with TPN after cystectomy [5]. Despite the current lack of data demonstrating the impact of EEN in reducing the incidence of postoperative ileus, the time needed to resume a full diet, the length of hospital stay, and the considerable economic and clinical benefits associated with EEN support its routine use after cystectomy, in agreement with enhanced recovery after surgery (ERAS) protocols [5].

Among the formulas for both oral and enteral use, those enriched with specific nutrients offer an additional immune-nutritional boost. The term “immunonutrition” (IMN) refers to specific substrates such as omega-3 fatty acids, arginine, glutamine, and nucleotides, which are able to upregulate host immune response, modulate inflammatory response, and improve protein synthesis after surgery [28]. In fact, after surgery, the inflammatory reaction may damage skeletal muscle tissue and induce an immunosuppressed state that increases the susceptibility to infections [1,28]. Many studies have highlighted that perioperative IMN reduces both the postoperative infection rate and length of hospital stay in major surgical settings [29,30]. Accordingly, ESPEN guidelines recommend the peri- or, at least, postoperative provision of IMN for malnourished patients undergoing major cancer surgery, with immune-modulating ONS to be administered for five to seven days preoperatively [31].

While a large mass of data is available on the efficacy of perioperative IMN in gastric, colorectal, and pancreatic cancer surgery patients [32,33,34], data regarding the benefit of perioperative IMN in patients with urological cancer undergoing surgical interventions are scarce due to the small sample size of the available studies [35]. Furthermore, the role of IMN within enhanced recovery pathways remains unclear as the meta-analyses on this topic were performed before the implementation of ERAS protocols [36].

A previous review showed promising data supporting the use of preoperative IMN [37]. Therefore, the aim of this narrative review is to describe the state of the art of the available scientific literature with additional data in the context of IMN in RC for cancer and to suggest possible future perspectives to be explored.

## 2. Materials and Methods

The PubMed electronic database updated until April 2023 was reviewed using the following keywords: “immunonutrition” AND “bladder cancer” OR “cystectomy” OR “urology”.

All articles were manually checked to select only appropriate scientific articles (prospective, retrospective, and case–control studies, and randomized clinical trials) and remove duplicate and non-English records. Moreover, the references of the included articles were also checked for the identification of additional relevant studies.

## 3. Results

Overall, nine studies conducted with patients receiving IMN before bladder surgery were retrieved and systematically reviewed (Table 1). The few available studies showed wide heterogeneity in terms of the nutritional data collected and the study endpoints (Table 2 and Table 3).

The pilot study of Bertrand et al. was the first to evaluate postoperative complications in a consecutive prospective group of patients receiving preoperative IMN (three IMN cartons/day for 7 days, 87% compliance rate) before RC and to compare the results with a retrospective matched group without IMN. The authors observed a lower rate of postoperative complications (40% vs. 77%, *p* = 0.008), reduced antibiotic use (23% vs. 60%, *p* = 0.008), lower incidence of paralytic ileus at day 7 (7% vs. 33%, *p* = 0.002), lower rate of pyelonephritis (17% vs. 47%, *p* = 0.003), and an overall length of stay reduction of 3 days (*p* = 0.51) in the IMN group [38]. 

Subsequently, Hamilton-Reeves registered a pilot randomized controlled trial (NCT01868087) aiming to determine whether an IMN-enriched supplement administered before and after RC surgery favors a reduction surgical complications. From this project, two studies were published. The first addressed the impact of IMN before (three IMN cartons/day for 5 days, 71% compliance rate) and after (three IMN cartons/day for 5 days, 86% compliance rate) RC on immune response and infection rates. Compared with patients supplemented with standard ONS, those receiving IMN had a lower rate of infection in the first 90 days post surgery (−39%, *p* = 0.027), lower levels of neutrophil-to-lymphocyte ratio 3 h after the first incision (*p* = 0.039), and fewer total myeloid-derived suppressor cells on postoperative day 2 (*p* < 0.001) [39].

The secondary analysis focused on the impact of perioperative IMN on the Th1–Th2 balance, interleukin (IL)-6 concentration, and nutritional status. The authors reported a favorable shift in Th1–Th2 balance preoperatively (+54% vs. −5%, *p* = 0.027) and a 43% reduction in IL-6 levels in the IMN group on postoperative day 2 (*p* = 0.020). Moreover, in the IMN group, the plasma arginine was stable from baseline to postoperative day 2, while in the ONS group, a 26% reduction from baseline to postoperative day 2 occurred (*p* = 0.0003). In terms of body composition, a trend toward reduced muscle loss in patients receiving IMN compared to those receiving standard ONS was observed at postoperative day 14 (7% vs. 17%, *p* = 0.078) [40].

Between the two previous pilot studies, Lyon and colleagues investigated the role of preoperative supplementation with high-arginine IMN (four IMN cartons/day for 5 days, 83% compliance rate) before RC. The IMN supplements were well tolerated, and their administration was safe. Interestingly, the reasons for incomplete supplement intake were also investigated, including volume oversaturation, nausea, and forgetfulness. However, no differences were detected in terms of infectious and non-infectious complications rate, postoperative length of stay, and readmission rate (all *p* > 0.4) compared to a retrospective cohort of untreated patients [41].

Compliance to IMN supplementation was also tested by Kukreja and coworkers, who published their results as an abstract only. Perioperative supplementation based on a novel IMN regimen (arginine and omega-3 fatty acids for 5 days before and 14 days after RC) was considered acceptable and tolerable by patients, with 76% of the prescribed dose being consumed [42].

In 2019, Ritch and colleagues compared the effects of IMN-enriched ONS (two IMN cartons/day for 3–4 weeks before RC and 4 weeks after, 88% compliance rate) to standard ONS. Specifically, they evaluated the efficacy on body composition assessed by imaging, serum biomarkers, nutrient intakes, inpatient and post-discharge complications, readmission rates, and mortality after RC. While no significant differences were detected in the change of total energy intake between the two groups, weight loss (−5 vs. −6.5 kg, *p* = 0.04) and muscle mass loss (−5 vs. −3.2 cm^2^/m^2^, *p* = 0.01) significantly differed in favor of IMN. Remarkably, the proportion of sarcopenic obese patients decreased by 33% in the IMN group, while it increased by 17% in the standard group (*p* = 0.01). Conversely, the length of stay and 30-day hospital-free days were similar in the two groups. A lower rate of overall complications (19% vs. 25%, *p*-value not reported) and a lower readmission rate (7% vs. 17%, *p* = 0.17) were reported in favor of IMN, although statistical significance was not reached [43].

In 2021, two research groups published the results of retrospective analyses in the context of ERAS, which failed to observe IMN advantages, unlike previous prospective studies. Cozzi and coworkers evaluated the incidence of surgical complications in patients who received perioperative IMN (three IMN cartons/day for 7 days before RC and two/day for 7 days, 88% had an adherence < 80%) compared with no supplementation. The study showed a higher infection rate (38% IMN vs. 8% control, *p* = 0.009) and readmission rate (15% IMN vs. 0% control, *p* = 0.03) in the experimental arm. The IMN group had significantly lower rates of urinary tract infections (8% vs. 19%, OR 0.4, 95% CI [0.13–0.9], *p* = 0.02) and *C. difficile* colitis (3% vs. 12%, 95% CI [0.04–0.8], *p* = 0.015). The use of TPN was not different between groups (23% IMN vs. 15% control, *p* = 0.48). With regard to the compliance rate, 92% of patients consumed all IMN volume before RC, and 88% had an adherence < 80% after surgery [44]. Finally, Khaleel and colleagues investigated the role of IMN on postoperative outcomes and length of stay in a large series of patients who received IMN before RC (one IMN carton/day for 5 days) and compared the outcomes to a matched group who did not receive IMN supplementation. The IMN group had significantly lower odds of requiring postoperative TPN (17% vs. 36%; OR 0.4, 95% CI [0.2–0.9], *p* = 0.015) and developing postoperative infection (25% vs. 45%, OR 0.4, 95% CI [0.2–0.8], *p* = 0.003), but no significant differences in other outcomes were detected. Preoperative albumin levels and body mass index (BMI) were not different between the two groups [45].

In 2022, in the setting of an ERAS-based protocol, a case–control study compared an historical cohort to a group treated preoperatively with IMN (three IMN cartons/day for 5 days before surgery) associated with carbohydrate load (maltodextrin) the night before surgery; no differences in infectious complication rate (43% vs. 37%, *p* = 0.53) and readmission rate within 30 days (22% vs. 15%, *p* = 0.34) were detected [46]. However, the return of bowel function occurred earlier in the IMN group than in the control group (3.12 days vs. 3.74 days, RR 0.82, 95% CI [0.7–0.9], *p* = 0.003).

## 4. Discussion

ESPEN guidelines recommend the provision of IMN specifically to malnourished patients undergoing major oncological surgery [31]. In the context of bladder surgery, there is no consensus on the optimal tool for identifying malnutrition. Several approaches are commonly used for screening and assessing malnutrition, including the Global Leadership Initiative on Malnutrition (GLIM) criteria [47], the Subjective Global Assessment (SGA) tool [48], and the criteria set forth by the Academy of Nutrition and Dietetics and the American Society for Parenteral and Enteral Nutrition (AND/ASPEN) [49]. According to the Global Leadership Initiative on Malnutrition (GLIM) criteria, the evaluation of phenotypic criteria (non-volitional weight loss, low BMI, and reduced muscle mass) and etiologic criteria (reduced food intake or assimilation, and inflammation or disease burden) is needed for the diagnosis of malnutrition, but screening of nutritional risk remains a mandatory step [47]. Originally designed to assess poor surgical outcomes, the SGA tool combines the patient’s medical history (including weight changes, dietary intake, gastrointestinal symptoms, functional capacity, and metabolic stress) with a physical examination to identify the presence of fat loss, muscle wasting, and fluid imbalances [48]. The scored Patient-Generated Subjective Global Assessment (PG-SGA) form has been validated for assessing the nutritional status of cancer patients [50]. The consensus statement by AND/ASPEN provides standardized guidelines for identifying and documenting malnutrition based on specific characteristics. These include unintentional weight loss, evidence of inadequate intake, muscle loss, subcutaneous fat loss, fluid accumulation, and diminished functional status, as measured by handgrip strength [49].

Nutritional risk was not described in the majority of the reviewed papers, and no study reported the use of GLIM criteria for malnutrition diagnosis. The presence of cancer requiring major surgery can be considered an etiologic criterion for nutritional risk, which has already been met. Conversely, anthropometric, body composition, and food intake data should be routinely collected as an integral part of the multidisciplinary assessment of cancer patients in order to define the presence of malnutrition, or at least to define malnutrition risk according to validated screening tools [24].

This narrative review aimed to describe the state of the art of the available scientific literature in the context of IMN in radical cystectomy. Some available results are promising and mainly consistent for a positive effect, regardless of nutritional status, suggesting that nutritional support with IMN goes beyond the importance of correcting nutritional derangements. Conversely, the findings of Cozzi [44] and Khaleel [45] are not in agreement with the previous ones. The use of ERAS protocols after RC is supported by significant literature [51], but the description of nutritional interventions within the context is often not made explicit. Moreover, the retrospective nature of some studies predisposes to bias of recall or classification, reduced identification of confounding factors, and difficulties in the assessment of causal and temporal relationships. Furthermore, the arbitrariness of inclusion criteria selection could affect the sample characteristics that may be related to the efficacy of nutritional interventions and, consequently, to the significance of the results taken as whole. For example, the study by Bertrand et al. excluded patients unable to take oral feeding [38], and that by Ritch and colleagues excluded those with dietary restrictions or food allergies precluding the consumption of supplementation [43]. Otherwise, Hamilton-Reeves’ studies excluded malnourished patients according to weight loss and BMI criteria [39,40]. Two recent reviews [52,53] assigned a low quality of evidence to some of the described studies due to imprecision errors and small sample size. Unfortunately, the differences in terms of study design, clinical endpoints, sample size, timing of nutritional intervention, and type of immunonutrition supplements do not enable a proper comparison of the results or to draw solid conclusions.

Indeed, high-quality trials characterized by a more rigorous standardization of intervention protocols are required. DC is the first strategy to ameliorate oral food intake in malnourished patients and those at nutritional risk, but it also represents a good opportunity to improve the compliance rate with oral nutritional supplements. Moreover, DC enables the provision of evidence-based nutrition education, which helps cancer patients to “empower” themselves as active and autonomous participants in the nutritional care process [14]. Nonetheless, the implementation of tailored enteral nutrition protocols for post-operative care should be considered as well.

Lastly, muscle mass is essential for the body’s defense mechanisms and immune function, and during acute stress like surgery or infection, protein breakdown increases due to increased metabolic demands. Exercise shows promise as a non-pharmacological approach to combat muscle wasting by reducing inflammation and promoting muscle growth and function. This can lead to improved postoperative recovery, reduced risk of complications, and enhanced response to acute stressors [54]. However, further research is necessary to identify the most effective strategies that integrate nutritional interventions and exercise programs for optimizing patient outcomes in the context of bladder surgery.

## 5. Conclusions

Reduced food intake, increased muscle catabolism, and inflammation are key factors in the etiology of cancer-related malnutrition and promote the deterioration of nutritional status and immunological competence. Although the role of IMN is still little explored in the context of bladder cancer surgery, the preliminary available data are promising and suggest that IMN may improve postoperative outcomes after RC through immunomodulation. The development of well-designed randomized control trials is needed to prospectively collect uniform and comparable data without confounding or selection biases in order to evaluate the effectiveness of IMN—regardless of the presence of malnutrition—and to identify the most suitable dosage, timing, and duration of support.

## Figures and Tables

**Table 1:** Reviewed studies on IMN intervention in the perioperative setting for RC.

1st Author Year, Country	Design	Sample Size, Males (%)	Age (Mean ± SD) or (Median (Range)), Years	Histology Neoadjuvant Chemotherapy (Yes/Sample)	Intervention	Type of IMN [Immuno-Nutrients]	Significant Results (IMN vs. ONS)
Bertrand J 2014, France [38]	Prospective Multicenter, pilot case–control study	60 30 IMN, 77% 30 CG, 83%	IMN: 70 (52–85) CG: 69 (50–89)	Bladder carcinoma 4/30 (both IMN and CG)	3 IMN cartons/day for 7 d before RC vs. retrospective, matched CG without IMN	Oral Impact^®^ Nestlé Health Science [arginine, nucleotides, omega-3 fatty acids]	Postoperative complications: 40% vs. 77%, *p* = 0.008; Antibiotic use: 23% vs. 60%, *p* = 0.008; Paralytic ileus at day 7: 7% vs. 33%, *p* = 0.002; Pyelonephritis: 17% vs. 47%, *p* = 0.003; LOS: −3 days (overall), *p* = 0.51; The compliance rate in the IMN group was 87%.
Hamilton-Reeves JM 2016, USA [39]	Prospective Pilot RCT	29, 100% 14 IMN 15 ONS	IMN: 70 ± 7 ONS: 68 ± 8	Bladder carcinoma 7/14 4/15	3 IMN cartons/day vs. 3 ONS (Boost Plus^®^) cartons/day for 5 d before and 5 d after RC	Impact Advanced Recovery^®^ Nestlé Health Science [arginine, omega-3 fatty acids, vitamin A, nucleotides]	IMN group compared to ONS: MDSC was lower 2 d after RC (*p* < 0.001); NLR was lower 3 h after the first incision (*p* = 0.039); Postoperative complications at 90 d: 14% vs. 47%, *p* = 0.06; Infection rate: −39%, *p* 0.027; 71% (10/14) reported that they consumed all IMN cartons before surgery; 86% (12/14) resumed supplementation within 24 h of surgery.
Lyon TD 2017, USA [40]	Prospective Phase II pilot study + retrospective control group	144 40 IMN, 72% 104 CG, 70%	IMN: 70 (63–78) CG: 69 (61–76)	Not specified 6/40 9/104	4 IMN cartons/day for 5 d before RC vs. CG without IMN	Impact Advanced Recovery^®^ Nestlé Health Science [arginine, omega-3 fatty acids, vitamin A, nucleotides]	83% of patients consumed all prescribed volume; No serious adverse events were reported.
Hamilton-Reeves JM 2018, USA [41]	Prospective Pilot RCT	29, 100% 14 IMN 15 ONS	IMN: 70 ± 7 ONS: 68 ± 8	Bladder carcinoma 7/14 4/15	3 IMN cartons/day vs. 3 ONS (Boost Plus^®^ Nestlé Health Science) cartons/day for 5 d before and 5 d after RC	Impact Advanced Recovery^®^ Nestlé Health Science [arginine, omega-3 fatty acids, vitamin A, nucleotides]	Th1-to-Th2 ratio: +54% vs. −5%, *p* = 0.027; IL-6: 43% lower in the IMN group on POD2, *p* = 0.020; Arginine: reduction of 26% from baseline to POD2 in the ONS group, *p* = 0.0003.
Kukreja JB 2019, USA [42]	Prospective Pilot RCT	46, gender not specified 23 IMN 23 CG	Not specified	Not specified Not specified	Arginine supplement (120 mL/day) + omega-3 fatty acids capsules (4 g/day) for 5 d before and 14 d after RC CG intervention was not reported	Not specified [arginine, omega-3 fatty acids]	76% of dose was consumed.
Ritch CR 2019, USA [43]	Prospective Pilot RCT	61 31 IMN, 84% 30 ONS, 93%	Median 68 IMN: 69 ONS: 67	Urothelial bladder carcinoma	2 IMN cartons/day vs. 2 ONS (Member’s Mark^®^ Multivitamin) cartons/day for 3–4 w before and 4 w after RC	Ensure^®^ Clinical Strength [omega-3 fatty acids, b-hydroxy b-methyl butyrate]	WL: −5 vs. −6.5 kg, *p* = 0.04; Muscle mass loss: −5 vs. −3.2 cm^2^/m^2^, *p* = 0.01; Sarcopenic obesity: −33% vs. −17%, *p* = 0.01. The compliance rate was 88% in IMN group.
Cozzi G 2021, Italy [44]	Retrospective study	52 26 IMN, 81% 26 CG, 81%	IMN: 68 (57–71) CG: 68 (63–71)	Not specified 11/26 12/26	3 IMN cartons/day for 7 d before RC + 2/day for 7 d postoperatively vs. CG without IMN	Oral Impact^®^ Nestlé Health Science [arginine, nucleotides, omega-3 fatty acids]	Documented infections: 38% vs. 8%, *p* = 0.009; Readmission rate: 15% vs. 0%, *p* = 0.03; 92% of patients consumed all IMN volume before RC. After, 88% had an adherence < 80%.
Khaleel S 2021, USA [45]	Retrospective study	204, 76% 104 IMN 100 CG	69 (60–75)	Bladder cancer 93/204	1 IMN carton/day for 5 d before RC vs. CG without IMN	Impact^®^ Nestlé HealthCare Nutrition [arginine, nucleotides, omega-3 fatty acids]	Postoperative TPN: 17% vs. 36%, *p* = 0.015; Postoperative infections: 25% vs. 45%, *p* = 0.003); Urinary tract infections: 8% vs. 19%, *p* = 0.02; *C. difficile* colitis: 3% vs. 12%, *p* = 0.015.
Patel SY 2022, USA [46]	Retrospective Case–control study	170 78 IMN, 90% 92 CG, 85%	IMN: 71 (43–87) CG: 71 (41–89)	Not specified	3 IMN cartons/day for 5 d before surgery + maltodextrin the night before surgery and 2 h prior to surgery vs. CG without IMN	Not specified [arginine, nucleotides, omega-3 fatty acids]	Return of bowel function: 3.12 d vs. 3.74, *p* = 0.003; The compliance was 100%.

Abbreviations: CG, control group; d, days; h, hours; IL, interleukin; IMN, immunonutrition; LOS, length of stay; n, number; MDSC, myeloid-derived suppressor cells count; NLR, neutrophil-to-lymphocyte ratio; ONS, standard oral nutritional supplement; POD, postoperative day; RC, radical cystectomy; RCT, randomized controlled trial; SD; standard deviation; Th, T-helper; vs., versus; TPN, total parenteral nutrition; w, weeks; WL, weight loss.

**Table 2:** Nutritional status parameters included in the selected studies.

1st Author Year, Country	BMI	Weight Loss	Muscle Mass	Food Intake	Compliance Rate to IMN	Nutritional Risk Screening or Nutritional Assessment
Bertrand J 2014, France [38]	/	/	/	/	yes	/
Hamilton-Reeves JM 2016, USA [39]	yes	yes	/	yes	yes	PG-SGA
Lyon TD 2017, USA [40]	yes	/	/	/	yes	/
Hamilton-Reeves JM 2018, USA [41]	yes	yes	DXA	yes	yes	PG-SGA
Kukreja JB 2019, USA [42]	/	/	/	/	yes	/
Ritch CR 2019, USA [43]	yes	yes	CT, DXA	yes	yes	/
Cozzi G 2021, Italy [44]	yes	yes	/	/	yes	MUST
Khaleel S 2021, USA [45]	yes	/	/	/	/	/
Patel SY 2022, USA [46]	yes	/	/	/	yes	/

Abbreviations: BMI, body mass index; CT, computed tomography; DXA, dual energy X-ray absorptiometry; IMN, immunonutrition; MUST, malnutrition universal screening tool; PG-SGA, patient-generated subjective global assessment.

**Table 3:** Aims of the included studies.

1st Author Year, Country	Aim
Bertrand J 2014, France [38]	To evaluate postoperative complications in a consecutive prospective group of patients receiving preoperative IMN and to compare the results with a retrospective matched group without IMN.
Hamilton-Reeves JM 2016, USA [39]	To evaluate the efficacy of IMN on immune response and infection rates in men consuming either IMN or standard ONS before and after RC.
Lyon TD 2017, USA [40]	To investigate the effect of preoperative high-arginine IMN supplementation prior to RC and to compare it to historical controls.
Hamilton-Reeves JM 2018, USA [41]	To evaluate the impact of perioperative IMN intake on the Th1–Th2 balance, IL-6 concentration, and nutritional status compared to ONS controls.
Kukreja JB 2019, USA [42]	To test a novel IMN regimen and to investigate its acceptability and tolerability in the perioperative period.
Ritch CR 2019, USA [43]	To evaluate the effects of a IMN enriched ONS vs. standard ONS on body composition, serum biomarkers, nutrient intakes, inpatient and post-discharge complications, readmission rates, and mortality after RC.
Cozzi G 2021, Italy [44]	To report the incidence of surgical complications in patients who received perioperative IMN compared with retrospective controls who did not, and to investigate factors associated with complications.
Khaleel S 2021, USA [45]	To evaluate the role of IMN on postoperative outcomes and LOS in a large series of bladder cancer patients who received IMN prior to RC and compare their outcomes to a matched group who did not receive IMN.
Patel SY 2022, USA [46]	To investigate the effects of pre-operative IMN and carbohydrate loading on perioperative and recovery outcomes.

Abbreviations: IL, interleukin; IMN, immunonutrition; LOS, length of stay; ONS, oral nutritional supplement; RC, radical cystectomy; Th, T-helper; vs., versus.
